# Laccase-Treated Polystyrene Surfaces with Caffeic Acid, Dopamine, and L-3,4-Dihydroxyphenylalanine Substrates Facilitate the Proliferation of Melanocytes and Embryonal Carcinoma Cells NTERA-2

**DOI:** 10.3390/ijms25115927

**Published:** 2024-05-29

**Authors:** Hanluo Li, Martin Wilhelm, Christina Marie Baumbach, Michael C. Hacker, Michael Szardenings, Klaus Rischka, Andreas Koenig, Ellen Schulz-Kornas, Florian Fuchs, Jan Christoph Simon, Bernd Lethaus, Vuk Savković

**Affiliations:** 1 National “111” Center for Cellular Regulation and Molecular Pharmaceutics, Hubei Provincial Key Laboratory of Industrial Microbiology, Sino-German Biomedical Center, Hubei University of Technology, Wuhan 430068, China; lihanluo@hbut.edu.cn; 2 Department of Cranial Maxillofacial Plastic Surgery, University Hospital Leipzig, 04103 Leipzig, Germany; bernd.lethaus@medizin.uni-leipzig.de; 3 Department of Ear, Nose and Throat Diseases, and Head and Neck Surgery, University of Greifswald, 17475 Greifswald, Germany; martin.wilhelm@uni-greifswald.de; 4 Julius-Bernstein-Institute of Physiology, Martin-Luther-University of Halle-Wittenberg, 06108 Halle, Germany; christina-marie.baumbach@uk-halle.de; 5 Institute of Pharmaceutic Technology and Biopharmaceutics, Department of Pharmacy, Math.-Nat. Faculty, Heinrich-Heine-Universität Düsseldorf, 40204 Düsseldorf, Germany; michael.hacker@hhu.de; 6 Institute for Pharmacy, Faculty of Medicine, Leipzig University, Eilenburger Straße 15 A, 04317 Leipzig, Germany; 7 Fraunhofer Institute for Cell Therapy and Immunology IZI, 04103 Leipzig, Germany; michael.szardenings@izi.fraunhofer.de; 8 Fraunhofer Institute for Manufacturing Technology and Advanced Materials IFAM, 28359 Bremen, Germany; klaus.rischka@ifam.fraunhofer.de; 9 Polyclinic for Dental Prosthetics and Material Sciences, University Hospital Leipzig, 04103 Leipzig, Germany; akoenig@uni-leipzig.de (A.K.);; 10 Department of Cariology, Endodontology and Periodontology, University Hospital Leipzig, 04103 Leipzig, Germany; ellen.schulz-kornas@medizin.uni-leipzig.de; 11 Clinic for Dermatology, Venereology and Allergology, University Hospital Leipzig, 04103 Leipzig, Germany; jan-christoph.simon@medizin.uni-leipzig.de

**Keywords:** cell culture, melanocyte, NTERA-2, laccase surface modification, adherence, proliferation

## Abstract

This study presents the effects of treating polystyrene (PS) cell culture plastic with oxidoreductase enzyme laccase and the catechol substrates caffeic acid (CA), L-DOPA, and dopamine on the culturing of normal human epidermal melanocytes (NHEMs) and human embryonal carcinoma cells (NTERA-2). The laccase–substrate treatment improved PS hydrophilicity and roughness, increasing NHEM and NTERA-2 adherence, proliferation, and NHEM melanogenesis to a level comparable with conventional plasma treatment. Cell adherence dynamics and proliferation were evaluated. The NHEM endpoint function was quantified by measuring melanin content. PS surfaces treated with laccase and its substrates demonstrated the forming of polymer-like structures. The surface texture roughness gradient and the peak curvature were higher on PS treated with a combination of laccase and substrates than laccase alone. The number of adherent NHEM and NTERA-2 was significantly higher than on the untreated surface. The proliferation of NHEM and NTERA-2 correspondingly increased on treated surfaces. NHEM melanin content was enhanced 6-10-fold on treated surfaces. In summary, laccase- and laccase–substrate-modified PS possess improved PS surface chemistry/hydrophilicity and altered roughness compared to untreated and plasma-treated surfaces, facilitating cellular adherence, subsequent proliferation, and exertion of the melanotic phenotype. The presented technology is easy to apply and creates a promising custom-made, substrate-based, cell-type-specific platform for both 2D and 3D cell culture.

## 1. Introduction

Cultivation of mammalian cells in vitro largely depends on their capacity to attach to the polymer surfaces of cell culture plastic, based on cell–substrate adhesion. Cells adhere to surfaces via a highly dynamic and complex interaction of cell adhesion molecules (CAMs) on their cell membrane [1,2] and the negatively charged substrate surface, with a particularly high affinity towards solid–liquid interfaces [3]. CAMs on the cell membrane include integrins, selectins, cadherins, and members of the immunoglobulin superfamily (IgSF) [4,5]. Regulation of in vitro cell adhesion involves signaling cascades that also regulate cell cycle, differentiation, migration, and survival [1]. Duration of the adherence processes varies depending on particular cell lines, cell types, and adherence properties of the surface [6]. Upon successful adherence, the cells increase their contact surface and begin to proliferate. Therefore, the cell adherence and adherent properties of the cell culture vessel surfaces present a basic technical requirement for static in vitro cultivation [7].

Polystyrene (PS) has been employed as a prime material for mammalian cell culture for the past five decades. PS is a polymer processed by injection molding, easily sterilized, and with excellent optical properties [8,9]. Nevertheless, the drawbacks of PS—weak hydrophilic properties and lack of surface chemistry for cell anchoring—may affect cell attachment, proliferation, and movement [10].

Improvements in PS surface chemistry, wettability, and hydrophilic properties have mainly been introduced by increasing the number of surface hydroxyl groups and reducing carboxyl groups via oxidation techniques [11], most commonly by plasma treatment [12]. The common reaction mechanism of both atmospheric and vacuum plasma surface treatment methods is based on the formation of reactive oxygen species as a result of reactions between the active plasma and PS, which causes an increase in oxygen content in the material and thereby a reduction in aromatic groups. Importantly, the phenolic and carboxyl groups produced by the reaction can facilitate the transition of hydrophobic material to hydrophilic. The increase in wettability and roughness of PS enhances the surface protein affinity and supports the attachment and growth of cells [8]. The downsides of plasma treatment are high production demands, byproducts of ROS, and non-selective modifications of the entire treated surface. Amid those downsides, plasma-treated PS usually serves the purpose of a plastic surface for cell culture as “good enough”. The yet unmet needs for treatment of PS surfaces include the following: inexpensive, non-toxic increase in its adherent properties, and customized shaping of the treated area (printability).

In this study, we employed an alternative method of PS modification mediated by laccase and optimized by its substrates—caffeic acid (CA), L-DOPA, and dopamine—to improve PS hydrophilicity and surface architecture for the purposes of cell cultivation.

Laccases (benzenediol: oxygen oxidoreductase, EC 1.10.3.2) are historically well-represented enzymes in industrial technology. Laccase was first isolated from the extract of the Japanese tree *Rhus vernicifera* [13] and can also be found in fungi, insects, and bacteria. It is a multi-copper enzyme complex that contains polyphenol oxidase with two enzyme active centers containing four copper ions [14]. The copper ions execute a critical function in enzymatic redox reactions, thereby catalyzing the oxidation of phenolic and aromatic substrates by receiving transferred electrons and reducing the molecular oxygen. Thus, during the process of enzymatic oxidation, the electrons are continuously transferred away from the reduced substrate and bound by oxygen to form water molecules [15].

Laccase thereby catalyzes the formation of unstable radicals from phenolic and aromatic substrates, facilitating their polymerization. Certain substrates can be oxidized by laccase through the additional involvement of mediating compounds, forming the laccase mediator system (LSM) as a result of the interaction between laccase, substrates, and mediator forms. The enzymatic redox reaction catalyzed by laccase provides a non-aggressive surrounding for lifeforms since it omits the generation of hydrogen peroxide, ROS, and quinone/semiquinone [16]. Thus, laccase has been widely applied in the degradation of lignin [17] and the treatment of hazardous pollutants [18].

Laccases (E.C. 1.10.3.2), vital lignolytic oxidoreductases, are ubiquitous in nature and oxidize a range of compounds, including environmental xenobiotics with challenging bioactivation. Thereby, the laccases perform crucial functions in a wide variety of organisms and present a base for diverse biotechnological applications. Laccase is crucial for bioremediation by degradation of toxic pollutants [19]. Enhancing their stability and reusability through immobilization is of great importance for sustainable development [20]. Microbial laccases are being further explored for the purposes of breaking down emerging environmental contaminants, while novel nano-immobilization techniques in biosensors enhance enzyme stability and specificity for real-time monitoring [21]. The versatility of laccase has, among other things, recently been reported in the degrading of antibiotics with *Bacillus* sp. *FNT*, enhancing lignocellulose breakdown, synthesizing polymers for food packaging, and decolorizing dyes using a hybrid nanobiocatalyst [19,22,23,24,25,26]. However, few studies have focused on laccase application on the interface treatments for improving surface chemistry, especially for cell cultivation.

The common, highly reactive substrates of laccase, i.e., the catechols, hydroxycinnamic caffeic acid (CA), dopamine, and its precursor L-DOPA, are capable of forming poly(catechol) compounds under the catalysis of laccase [18], which are also active as hydrophilic ligands [27]. The propensity of dopamine to spontaneously build polymers has been demonstrated by Lee and associates by dip-coating various surfaces with dopamine aqueous solution; such polymers included polystyrene (PS), polyethylene (PE), polycarbonate (PC), polyethylene terephthalate (PET), polytetrafluoroethylene (PTFE), polydimethylsiloxane (PDMS), polyetheretherketone (PEEK), and polyurethanes (Carbothane (PU1) and Tecoflex (PU2)). The resulting spontaneously formed multifunctional polymer coating appeared to be independent of the substrate composition [28].

In this study, in order to actively modify the PS surface for the purposes of improving its adherence properties by increasing their hydrophilicity and roughness on a µm-scale, laccase combined with its substrates was selected.

Laccases have by now already been used in the development of surface modification techniques. Urena et al. [29] modified carbon material by means of an aqueous suspension of laccase and maltodextrin and suggested that this laccase enzyme-based surface modification method could improve the wettability of carbon-based materials. Szardenings et al. also reported a method that utilizes oxidoreductase enzymes, including laccase, with soluble oxygen as a hydrogen receptor to oxidize the hydroxyl-substituted phenyl groups in the substrates. Thereby, the surface of the material was activated, increasing the hydrophilicity and bonding the target structures of proteins, cells, carbohydrates, peptides, and amino acids [28]. Accordingly, the laccase-based surface modification has already been identified as a candidate procedure for modifying cell culture plastic, including PS culture vessels.

To the best of our knowledge, PS modified as aforementioned has, to this day, not been employed for culturing cells with sensitive adherence properties, such as embryonic kidney cells [30], cancer stem cells, breast and colon cancer cell lines [31], embryonic carcinoma lines [32], or melanocytes [33]. For those reasons, we tested the modified PS in culture with normal human epidermal melanocytes (NHEMs) and the embryonic carcinoma cell line NTERA-2 [28]. To the best of our knowledge, this study is the first assessment of the laccase-based surface modification effects on those two cell lines [33,34,35,36]. NHEMs are well known for easily detaching in the process of differential trypsinization, and the adherence of NTERA-2 cells is low as in most embryonic carcinoma cell lines [37,38]. We assumed that the weak adherence of both cell types could be reinforced by activating the PS surface chemistry, which could subsequently corroborate cell proliferation. Since the proliferative properties of the chosen cell types are quite different—the NHEMs are slowly dividing cells, whereas the NTERA-2 proliferate rapidly [37,38]—this divergence enabled two different angles on the effect of surface modification on cell proliferation in two very different systems.

The melanocyte cell division and their melanogenesis (production of the pigment melanin) rarely stay in balance; one likely excludes the other and they happen non-simultaneously. Intensive proliferation is therefore usually a characteristic of amelanotic stages, whereas melanogenesis follows proliferation and marks a specialized, functionally differentiated status. As previously suggested, melanin precursors, DOPA, dihydroxyindole (DHI), and dihydroxyindole-2-carboxylic acid (DHICA) apparently suppress the growth of melanocytes, and they even exert cytotoxicity [39,40]. Clearly, the adherent surface balancing the conditions needed for both proliferation and melanogenesis in vitro would be of particular value for melanocyte cultivation. Based on this, we aimed at modifying the PS surface by laccase and its substrates CA, dopamine, and L-DOPA, expecting a change in surface structure (3D geometry), an optimized primary adherence, and subsequent enhanced proliferation of NHEM and NTERA-2, as well as a facilitated end phenotype of NHEM.

## 2. Results

### 2.1. Results

#### 2.1.1. Polymer Layer Formation by Laccase and Laccase–Substrate Treatments

**Figure 1:**
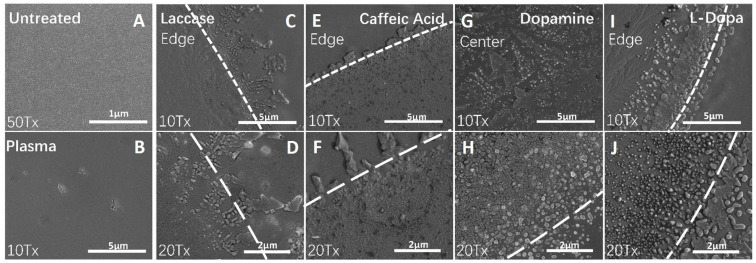
SEM of untreated, plasma-treated, and laccase–substrate-treated surfaces. Untreated, plasma, and laccase–substrate-treated surfaces were visualized by means of SEM. Different surface structures were revealed under high magnification. On untreated and plasma-treated PS, the surface was uniform and smooth. The laccase–substrate-treated surfaces showed an ample layer of insoluble substance, with clear edges distinguishable from the untreated surface (marked by the discontinuous white line). Depending on the substrate, different morphologies of substance layers were observed. (**A**) Untreated PS; (**B**) plasma-treated PS; (**C**) laccase-alone-treated PS, edge, magnification 10×; (**D**) laccase-alone-treated PS, edge, magnification 20×; (**E**) laccase–caffeic acid-treated PS, edge, magnification 10×; (**F**) laccase–caffeic acid-treated PS, edge, magnification 20×; (**G**) laccase–dopamine-treated PS, center, magnification 10×; (**H**) laccase–dopamine-treated PS, edge, magnification 20×; (**I**) laccase–L-DOPA-treated PS, edge, magnification 10×; (**J**) laccase–L-DOPA-treated PS, edge, magnification 20×.

PS surface modified by laccase and laccase–substrate was discernable as a circle with a clear edge (Figure 1). Untreated surfaces demonstrated a lack of polymer-like structures, even at higher magnification (Figure 1). On the laccase-treated surface, the compact alveolate layer was distinctly observed, with an unconfined crystalized and aggregated edge that could be generally characterized as polymer-like. The laccase–CA-treated surface was granular with a more distinct crystalized edge. The laccase–dopamine-treated surface displayed a non-confined circular layer with a thinner pattern. The layer generated by laccase–L-DOPA revealed a mixture of described morphologies, i.e., large granular structures and a non-confined edge.

**Figure 2:**
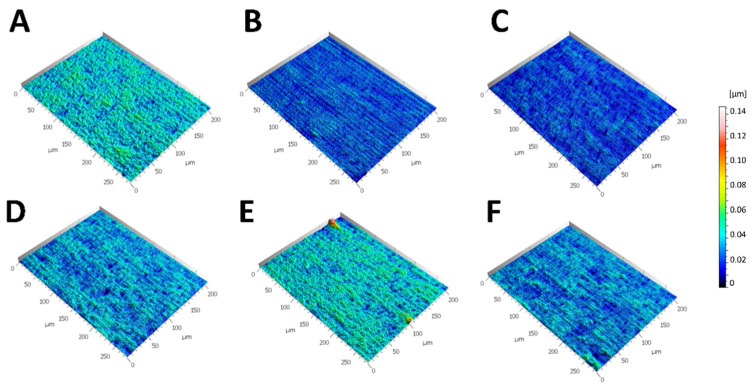
The 3D surface texture of untreated and treated surfaces. Three-dimensional surface textures in 45° lateral view; meshed axiomatic models filtered for the roughness component (S-L surface); (**A**) untreated PS; (**B**) plasma-treated PS; (**C**) laccase-treated PS; (**D**) laccase–caffeic acid-treated PS; (**E**) laccase–L-DOPA-treated PS; (**F**) laccase–dopamine-treated PS. Note that all surfaces are in the same z-scale. The x-axis and y-axis: horizontal distribution in µm; z-axis is displayed in color with color key on the right side.

The surface texture roughness gradient (*Sk*, core roughness depth) gradually decreased in accordance with the following substrate succession: L-DOPA > untreated > dopamine > caffeic acid > laccase, plasma (Figure 2). Thus, there is a gradual change in the surface geometry after treatment. Since the method works on a µm-scale, only µm changes are reported here. A detailed description of the measured surface texture features (Table 1) indicates which features of the surface geometry change in which aspect: the relationship of mean hill and mean dale area displayed similar size (*Sha*, *Sda*) and density (*Spd*, *Svd*) in all treatments. Yet, the peak curvature (*Spc*) was smallest in laccase and largest in laccase–dopamine-treated PS, whereas in other treatments it remained intermediate. The pit curvature (*Svc*) was the smallest on laccase-processed PS and the largest on plasma-treated PS. Plasma- and laccase-treated PS displayed the smallest hills and dale volumes (*Shv, Sdv*), as well as the smallest height/depth of hills and dales (*Spk*, *Svk*).

Notable changes were observed in the increase in mean hill area (general increase), mean hill volume (3-fold increase on L-DOPA-treated surfaces), reduced summit height (1.2-fold increase on dopamine-treated and 1.5-fold on L-DOPA-treated surfaces), mean dale area (increased on all treated surfaces, 2.5-fold increase on L-DOPA-treated surfaces), and mean dale volume (3-fold increase on L-DOPA-treated surfaces), as well as a decrease in peak density and pit density on all treated surfaces. No considerable changes were observed in roughness, peak curvature, and valley depth.

Judging by the heterogenic geometric structures of the surfaces observed in this study and taking into account the known catalytic mechanism of laccase and its polymerization effect in the presence of substrates, it was assumed that novel polymeric structures had been formed on the treated PS surfaces.

#### 2.1.2. Cell Adherence

**Figure 3:**
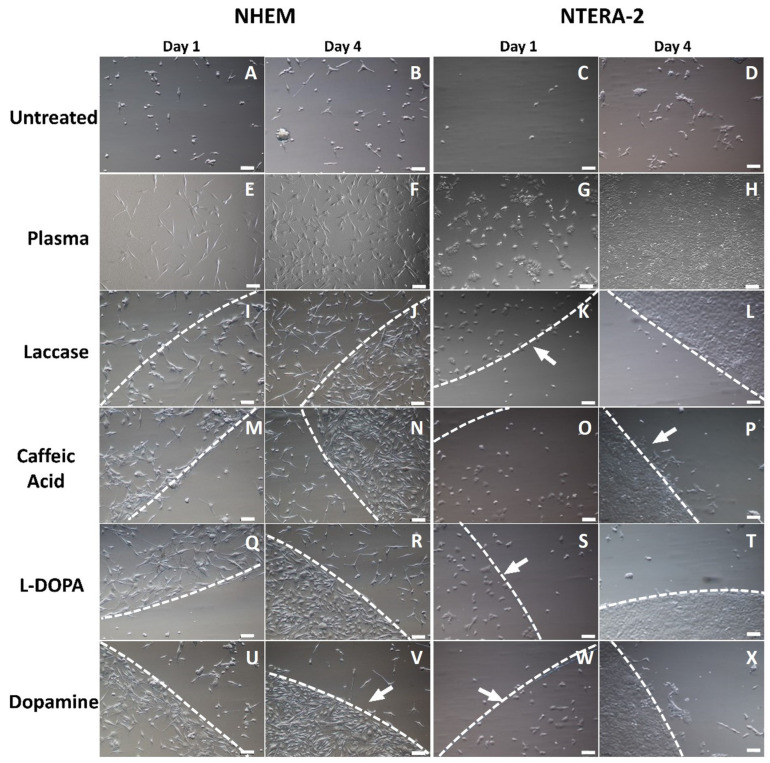
Cell attachment and proliferation of NHEM and NTERA-2 on unmodified and plasma-, laccase-, and laccase–substrate-modified surfaces on d1 and d4 after seeding. NHEM and NTERA-2 cells were seeded onto PS, intact or treated with laccase, laccase–CA, laccase–dopamine, and laccase–L-DOPA, and documented on d1 and d4. The difference in cell attachment between treated and untreated surfaces was clearly distinguishable, and the majority of cells were adherent in the treated areas with visible borders (borderline has been highlighted and marked with a discontinuous white line and an arrow). Bright field microscopy, 10× magnification, scale bar represents 100 µm. (**A**) Untreated PS, NHEM d1; (**B**) untreated PS, NHEM d4; (**C**) untreated PS, NTERA-2, d1; (**D**) untreated PS, NTERA-2, d4; (**E**) plasma-treated PS, NHEM d1; (**F**) plasma-treated PS, NHEM d4; (**G**) plasma-treated PS, NTERA-2, d1; (**H**) plasma-treated PS, NTERA-2, d4; (**I**) laccase-alone-treated PS, NHEM d1; (**J**) laccase-alone-treated PS, NHEM d4; (**K**) laccase-alone-treated PS, NTERA-2, d1; (**L**) laccase-alone-treated PS, NTERA-2, d4; (**M**) laccase–caffeic acid-treated PS, NHEM d1; (**N**) laccase–caffeic acid-treated PS, NHEM d4; (**O**) laccase–caffeic acid-treated PS, NTERA-2, d1; (**P**) laccase–caffeic acid-treated PS, NTERA-2, d4; (**Q**) laccase–L-DOPA-treated PS, NHEM d1; (**R**) laccase–L-DOPA-treated PS, NHEM d4; (**S**) laccase–L-DOPA-treated PS, NTERA-2, d1; (**T**) laccase–L-DOPA-treated PS, NTERA-2, d4; (**U**) laccase–dopamine-treated PS, NHEM d1; (**V**) laccase–dopamine-treated PS, NHEM d4; (**W**) laccase–dopamine-treated PS, NTERA-2, d1; (**X**) laccase–dopamine-treated PS, NTERA-2, d4.

NHEM and NTERA-2 cultivated on laccase-treated and laccase–substrate-treated surfaces showed normal, unaltered spindle morphology (Figure 3). One day, upon cell seeding (d1), cells preferentially attached to treated surfaces, even more so at d4, demonstrated by visible circular cell accumulation on treated areas, contrary to the untreated surfaces.

A distinct edge pattern was formed by both adherent NHEM and NTERA-2 cells along the border of the laccase-treated PS surface. Across this edge, cells adhered to the untreated surfaces to a much lower extent (Figure 3). At d4, NHEM confluence on the laccase-treated area reached 50–100% versus 50% on the plasma-treated and 10% on the untreated surface. In NTERA-2, the confluence at d4 reached 80–100% on laccase- and plasma-treated surfaces and 5% on untreated surfaces. The edge of the adherence zone on treated surfaces was more pronounced in the NTERA-2 culture.

On surfaces modified with caffeic acid, L-DOPA, and dopamine, the confluence of NHEM exceeded 90% on d4, whereas NTERA-2 reached 100% confluence on all modified surfaces. Contrary to that, only a small portion of NTERA-2 cells colonized the untreated surface, and clusters of dead NTERA-2 cells were observed.

#### 2.1.3. Cell Proliferation

The NHEM and NTERA-2 cells maintained typical general in vitro dynamics. After seeding on day 0 and a period necessary for adhering, a part of NHEM and a high quote of the NTERA-2 seeded cells adhered to the culture surface. During this period, the non-adherent cells could not survive, and the adhering cells were still not able to divide. First divisions occurred once the cells had assumed their typical morphology and reached a critical level of protein and DNA syntheses to make a transition to the M phase. Consequently, the number of adherent NHEM cells on day 1 was still lower than the total number of seeded cells, which appeared as a decrease in cell number in the diagram. The number of NTERA-2 on day 1 was comparable to that of day 0. After a short period of recuperation, the cells began to divide, thereby increasing their numbers on day 2 and further.

**Figure 4:**
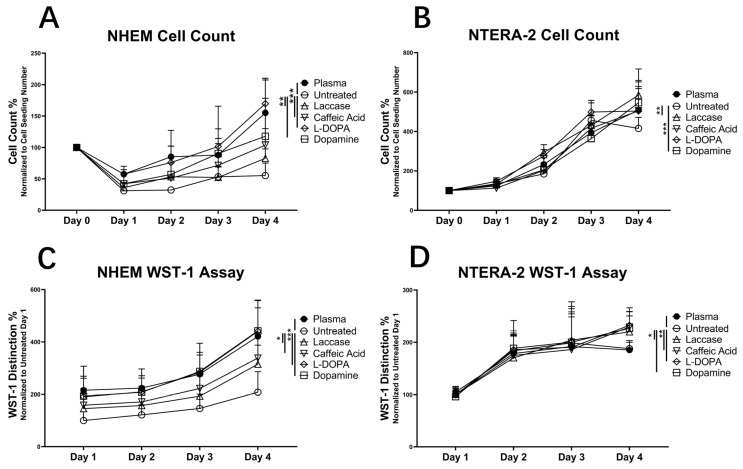
Cell count and WST-1 assay of NHEM and NTERA-2 cells. Direct cell count of NHEM (**A**) and NTERA-2 (**B**) on different surfaces over time. (**C**) NHEM WST-1 extinction standardized to the untreated surface extinction value (100%). (**D**) NTERA-2-WST-1 extinction standardized to the untreated surface extinction value (100%). Statistical significance at d4 (* *p* < 0.05, ** *p* < 0.01, *** *p* < 0.005). n = 3. The common normalized starting point for all cultures (100%) is marked with a black square.

NTERA-2 proliferated at a much higher rate than NHEM, as expected from the characteristics of the two cell types (Figure 4). The comparison of NTERA-2 and NHEM cell count on d1, d2, d3, and d4 with the initial seeding number of 30,000 cells on d0 revealed statistically significant differences between NTERA-2 and NHEM numbers on all days and on all surfaces (Figure 4A,B). 

Intrinsically low NHEM adherence resulted in a roughly 2-fold lower number of adherent cells on d1 vs. d0 (Figure 4). Despite this setback, NHEMs proliferated, in particular on the laccase-treated surfaces compared to the untreated. In NHEMs, on d4, the initially seeded 3 × 10^4^ cells were reduced to 16,556 ± 4871 on untreated PS, whereas they significantly increased to 46,500 ± 13,497 cells on the plasma-treated surface (2.8-fold, *p* = 0.00008), 24,777.67 ± 3695 on laccase-treated surface (1.5-fold, *p* = 0.0076), 31,111 ± 6431 on laccase–CA-treated surface (1.9-fold, *p* = 0.00066), 50,888.67 ± 9259 on the laccase–L-DOPA-treated surface (3-fold, *p* = 0.0000054), and to 35,334 ± 14,817 on laccase–dopamine-treated surface (2.1-fold, *p* = 0.0075).

**Figure 5:**
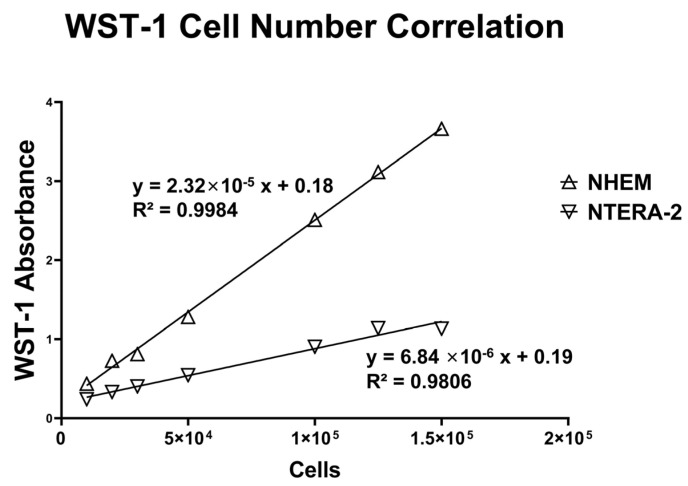
Correlation of cell number and WST-1-absorbance signal. In total, 3 × 10^4^ cells of NHEMs and NTERA-2 were seeded on PS. The direct cell count was determined by counting living cells in the Neubauer Chamber. Mitochondrial activity was measured by WST-1 assay on d1, d2, d3, and d4. Linear correlation of the cell number and the WST-1 signal in NHEM and NTERA-2 is depicted. Tested NHEM and NTERA-2 WST-1 extinction values were used for the standard curve (R^2^ (NHEM) = 0.9984, R^2^ (NTERA-2) = 0.9806, respectively) and compared with the values on plasma-treated PS.

The correlation between the cell number and the WST-1 absorbance signal is shown in Figure 5.

The number of adherent NTERA cells on d1 was comparable to the number of seeded cells, and they proliferated in the course of cultivation. On d4, the number of NTERA-2 cells was higher on the laccase-treated surface (175,333 ± 16,391.05 cells, 1.41-fold, *p* = 0.0058) and the laccase–dopamine-treated surface (163,889 ± 20,118 cells, 1.31-fold, *p* = 0.0007) than on the untreated surface (124,667 ± 13,714 cells).

The results of WST-1 assays corroborated the observation established by cell counting. Both NHEM and NTERA-2 cultured on surfaces treated with laccase or laccase–substrate showed significantly higher extinction of the WST-1 chromogenic outcome compared to untreated surfaces (Figure 4C,D). In NHEM, the extinction was significantly higher on surfaces modified with laccase (1.51-fold, *p* = 0.018), laccase–CA (1.62-fold, *p* = 0.018), laccase–L-DOPA (2.12-fold, *p* = 0.00019), and laccase–dopamine (2.12-fold, *p* = 0.00016) than in untreated samples. Analogous effects were quantified in NTERA-2, with a higher extinction on laccase- (1.17-fold, *p* = 0.02), laccase–CA- (1.22-fold, *p* = 0.003), laccase–L-DOPA- (1.24-fold, *p* = 0.004), and laccase–dopamine-treated surfaces (1.21-fold, *p* = 0.026) compared to untreated ones.

In short, the results of both the cell count and the WST-1 assay indicated an increased cell adherence as well as a subsequent increase in NHEM number and their activity at d4, corroborated by the cell density in culture on d1 and d4 (Figure 3). The untreated surfaces enabled lower cell adherence (Figure 3) and proliferation (Figure 4) compared to any of the modified surfaces, followed by that of the plasma-treated surface as the second lowest.

In the case of NHEMs, enhanced cell adherence was most intensively observed on surfaces modified with laccase–CA substrates. The cell numbers and WST conversion were enhanced significantly on the PS surfaces treated by laccase with L-DOPA and dopamine, compared to that of the untreated and the plasma-treated surface. Adhesion and proliferation on surfaces modified by laccase with L-DOPA and dopamine were higher compared to the modification by laccase alone (L-DOPA vs. laccase *p* = 0.005, dopamine vs. laccase *p* = 0.016) and laccase–CA (L-DOPA vs. CA *p* = 0.009, dopamine vs. CA *p* = 0.031).

In the case of NTERA-2, both adherence and proliferation of cells cultivated on all modified surfaces were higher than on the untreated surface with pronounced effects of CA, L-DOPA, and dopamine co-treatments on adherence and cell proliferation.

#### 2.1.4. Melanin Content in NHEMs

**Figure 6:**
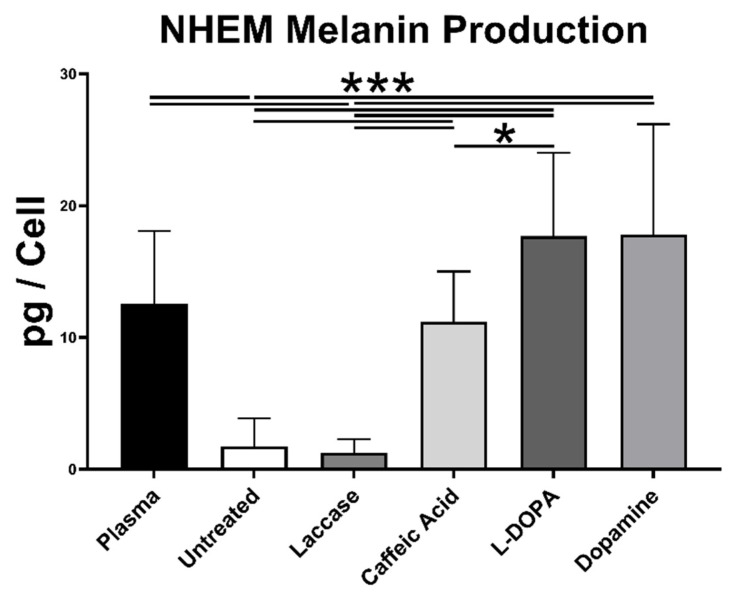
Melanin production in NHEM cells cultured on different surfaces. NHEM melanin production per cell on different surfaces. NHEM cultured on the laccase–CA-, laccase–dopamine-, and laccase–L-DOPA-treated surfaces displayed higher melanin production per cell than on the untreated surface. Melanin concentration was derived from the melanin standard with known concentration (not shown, R^2^ = 0.9971). Statistical significance (* *p* < 0.05, *** *p* < 0.005). n = 6.

The melanin content per cell, the final measure of melanocyte function, displayed significant variation between different surfaces (Figure 6). In comparison to the untreated surface, the melanin content per cell in NHEMs significantly increased when cultivated on the surface modified with laccase and CA, L-DOPA, and dopamine, respectively (6.42-fold for CA, *p* = 0.00017; 10.14-fold for L-DOPA, *p* = 7.71 × 10^−5^; 10.20-fold for dopamine, *p* = 0.00053) (Figure 6). Compared with NHEMs cultivated on laccase-treated PS, melanin production on surfaces co-treated with CA, L-DOPA, and dopamine was also enhanced (8.83-fold for CA, *p* = 5.18 × 10^−5^; 13.95-fold for L-DOPA, *p* = 4.36 × 10^−5^; 14.04-fold for dopamine, *p* = 0.00036). Plasma-treated surface sustained a 7.20-fold increase compared to the untreated surface (*p* = 0.00078).

### 2.2. Figures, Tables, and Schemes

Parameters included 3D surface roughness (*Sk*, core roughness depth) and six feature parameters describing the peaks/hills/summits (*Spd*, the density of peaks; *Spc*, arithmetic peak curvature; *Sha*, mean hill area; *Shv*, mean hill volume; *Spk*, reduced summit height) as well as the dales/valleys (*Svd*, the density of pits; *Svc*, arithmetic mean pit curvature; *Sda*, mean dale area; *Sdv*, mean dale volume; *Svk*, reduced valley depth) according to ISO 25178 [41] to illustrate 3D surface roughness. Results are shown as mean value ± SD. Notable changes were observed in peak geometry parameters: increase in mean hill area (general increase), mean hill volume (3-fold increase on L-DOPA-treated surfaces), reduced summit height (1.2-fold dopamine-treated and 1.5-fold L-DOPA-treated surfaces); increase in dale parameters: mean dale area (increased on all treated surfaces, 2.5-fold increase on L-DOPA-treated surfaces) and mean dale volume (3-fold increase on L-DOPA-treated surfaces); decrease in density parameters: peak density and pit density on all treated surfaces. No changes were observed in roughness, peak curvature, and valley depth.

## 3. Discussion

As an alternative to the plasma-mediated PS surface treatment, we increased PS adherent properties for the purposes of NHEM and NTERA-2 cultivation by modifying surface chemistry with laccase and polymerization of the catechol substrates CA, L-DOPA, and dopamine. These surface modifications enhanced PS compatibility with NHEMs and NTERA-2 in terms of their adherence, proliferation, and melanin content (NHEM) compared to the treatment with laccase alone, plasma-treated PS, and untreated hydrophobic PS.

The treated PS area was recognizable by the distinct edges between treated and untreated surfaces and the occurrence of novel polymer-like layers, insoluble and stable, with a granular, compact structure and characteristic patterns for each substrate. These layers suggest surface chemistry alterations and polymer formation, with likely greater hydrophilicity as expected from the earlier reports and with an evidently altered 3D surface roughness (see surface textures, Figure 2) [29]. Accordingly, the distinctly demarcated areas of laccase- and substrate-modified PS matched the area with the detected polymer-like pattern, and the cells at those areas displayed an improved cell attachment and proliferation. Contrary to this, the untreated surface did not show any structural change and provided only a modest level of cell attachment without a distinct pattern. Here, one could assume that there are already surface features present enabling modest cell attachment. Future control studies are needed to clarify the geometric limitations, e.g., at which scale of the surface geometry cells did not attach at all.

Modifications of the PS surface by laccase–dopamine and laccase–L-DOPA introduced the highest changes in geometric parameters of the surface (Figure 2, Table 1). A notable increase in the following parameters was observed: mean hill area (1.3-fold dopamine, 2.3-fold L-DOPA), mean hill volume (3-fold, L-DOPA), summit height (1.2-fold dopamine and 1.5-fold-DOPA), mean dale area (all treatments, 2,5-fold dopamine), and mean dale volume (3-fold, L-DOPA). These pronounced surface changes with dopamine and L-DOPA as substrates coincide with the sharpness of the demarcated edge between the modified and unmodified surfaces. According to the proliferation tests, laccase alone is capable of modifying the PS surface into a base that increases cell proliferation, both in NHEMs and NTERA-2. In the synergy of laccase and its substrates, dopamine and L-DOPA appear to provide the most potent surface change (Figure 1). Accordingly, laccase–dopamine- and laccase–L-DOPA-treated PS surfaces provided the fastest cell adherence (Figure 3) and the highest increase in cell proliferation of the NHEMs and NTERA-2 (Figure 4), as well as the highest and significantly increased melanin production in the NHEMs. (Figure 5). Here, we can discern a favorable surface geometric profile that may be a particularly good fit for melanocyte cell culture.

The modifications of the PS surface fit well with the previously reported data on the surface chemistry modifications by laccase and its substrates [29,42]. Here, both an increase in hydrophilicity as a consequence of enzyme adsorption and an augmented surface charge due to the polymerization with catechol substrates have been reported. Dopamine as a substrate results in a positive surface charge, CA in a negative charge, and L-Dopa in a zwitterionic charge [42]. The increased adherence of NHEMs and NTERA-2 followed by their proliferation and the NHEM melanin production can be generally interpreted as an effect of the increased hydrophilicity and the finely structured surface features (hills and dales), which also appear to be the core effect of the PS modifications performed in this study. Based on the aforementioned, we hypothesize that the treatment of PS by laccase and CA, L-DOPA, and dopamine as substrates resulted in the forming of a polymer layer, which increased core roughness depths and ionic charge of the surface, thereby intensifying the hydrophilicity and the subsequent interactions with CAM surface proteins of the cell membranes.

Hydrophilic surfaces have been shown to facilitate protein adsorption via their net charges and influence the selectivity and orientation of bound proteins [3,43]. In particular, three in vitro studies directly suggested that such enhanced hydrophilicity can promote the adsorption and preservation of vitronectin (VN) and fibronectin (FN), two extracellular adhesion proteins present in the serum-supplemented medium that have been identified as key players in the cell-surface attachment process [10,43,44].

At this instant, mechanisms of improving the PS surface chemistry and 3D surface texture features by laccase–substrate treatments can be interpreted as an effect of substrate polymerization under the catalyzing effect of laccases and binding of the polymerized substrates to the PS surface. This reliable, clean, and environmentally friendly process can be safely applied in cell culture, as shown here and elsewhere [15,18]. The absence of cell toxicity of the laccase-modified area in synergy with the applied substrates can be inferred from the readout of the undisturbed mitochondrial activity when compared to the untreated PS, and it is in accord with the aforementioned reported data.

The layers visualized in this study upon modification are interpreted as a result of the generally known mechanism of laccase-facilitated polymerization of 1,2-dihydrobenzol core-containing substrates such as CA, L-DOPA, and dopamine. Laccase by rule separates a hydrogen atom from the benzylic carbon–hydrogen bond at the p-site of the phenolic group and from arylamine, cleaving the alkyl side chain [42,45] carbon–carbon or carbon–oxygen bonds, forming unstable phenoxyl radicals at the opened ring ends and enabling polymerization via C-O-C, O-O, or C-C bonds at the phenolic group [16,46] o-site. Oxygen acts as both electron and proton receptor, and H atoms are oxidized to water. When substrates are added, the oxidation reaction yields intermediate quinones that can polymerize with substrate o-dihydroxyl groups, which leads to the non-toxic polymers attaching to the PS surface. In all probability, the SEM-documented layers found on PS upon modification with laccase, CA, L-DOPA, and dopamine are generated by this polymerization process.

All three substrates used in this study have been previously reported as being prone to polymerizing under the catalytic action of laccase, although through different modes of action. L-DOPA and dopamine can be polymerized into poly(catechol)s [42,46,47]. The resulting polymer layer has been reported to improve the roughness and wettability of untreated hydrophobic PS surfaces and thereby facilitate cell attachment [18], in accordance with our data. The oxidation reaction of laccase-catalyzed polymerization of the o-dihydroxyl catechols and the characterization of the polymers remains to be further investigated, as well as the potential of laccase alone, or of CA, L-DOPA, and dopamine alone, to improve the surface hydrophilicity of the untreated PS surface. In addition to hydrophilic improvement, CA is capable of reacting via its carboxy group, which may facilitate electrostatic bonds with Ca^2+^- and Mg^2+^ ions of cellular proteins, including integrin. This study did not directly focus on the specific link between the increased hydrophilicity introduced by laccase–substrate-mediated modifications of PS and the engagement of CAMs. Therefore, this particular aspect needs to be addressed in a separate future study.

This study has also clearly demonstrated direct causality between synergic surface modification by laccase and its substrates, successful adherence of melanocytes, and facilitated melanogenesis. The precise mechanism of the effects of laccase-mediated and polymerized CA, L-DOPA, and dopamine on melanogenesis has not been directly addressed here. Based on the previously reported data and corroborated by our results, the general explanation at hand is that melanocytes more easily adhere to such modified surfaces and in all probability act from a point of reinforced homeostasis, inherently using their spared resources for melanogenesis. Drastically enhanced mitochondrial activity of the NHEM, which stands out of proportion with their proliferation, speaks in favor of an increased metabolism and synthetic processes boosted by that advantageous resource status. However, the surplus in resources would unlikely create a base for the measured 6–14-fold increase in melanin production, especially in light of the contrast between production on laccase-treated and laccase–substrate-treated surfaces. This particularly stimulating effect of the laccase–substrate-generated polymers on NHEM melanogenesis suggests a link between adherence and melanogenesis. This correlation has been reported earlier, demonstrated as the effect of the most eligible adherence mediators laminin, integrin, and cadherins on melanin production [48]. Laminin regularly partakes in keratinocyte-mediated regulation of melanogenesis in melanocytes by controlling tyrosine uptake [49]. Integrins and cadherins are adherence-relevant proteins known to coordinate the migration of melanoblasts and their subsequent proliferation and differentiation into highly specialized melanotic melanocytes [50,51]. For those reasons, they are the most elicit candidates among the proteins affected by the applied surface changes. Accordingly, in our experimental setup, integrin may be additionally electrostatically bonded to the negative charge of the CA carboxy group, mediated by the Ca^2+^ and Mg^2+^ ions. These probable effects remain to be confirmed, and their exact mechanisms ought to be elucidated in succeeding studies.

Particular focus of this study was the biofunctionalization of the PS towards NHEM- and NTERA-2-friendly cell culture surfaces. Recent advances in laccase research focus on enhancing its applications through biotechnological innovations, particularly in bioremediation and biosensor development [21,24,52,53,54,55,56]. The potential of laccase on the surface treatment was used for improving the enzyme’s stability and efficiency through immobilization techniques [11]. This study explored the application of laccase on surface treatment for cell cultivation, benchmarking the conventional plasma treatment for the tissue culture surface.

Biofunctionalization of cell culture surfaces bears great significance in cell biology. Adherence is a prerequisite for all in vitro cell cultures. It enables the very survival of the cells and precedes their proliferation. The cell culture vessels are standardly produced en masse with a one-size-fits-all technological design that fits the basic requirements of most adhering cell lines. Nevertheless, the surfaces can be specifically adapted to the needs of a particular cell line by the means of biofunctionalization, as in this case, involving an enzymatic substrate that introduces a surface modification that is particularly favorable for specific cell line requirements. In the case of PS modifications by laccase and its substrates, two main trends can be clearly defined. One is the general support of the adherence and proliferation of both high-pace NTERA-2 and slow-pace-dividing NHEM cells, by all tested laccase–substrate-treated surfaces. The second is more specific, involving the potent effects of dopamine and L-DOPA as laccase substrates on surface modification, as well as enhanced proliferation of slow-dividing NHEMs and of their melanotic properties, which are supported by those surface modifications.

This can be of great importance in further mass production of cell culture adherence surfaces. The laccase–substrate method involves lower costs and higher efficiency in altering the PS surface into a better adherent and cell friendlier basis for culturing. On an industrial scale, this could mean serious cut-backs in terms of production costs, and on the bioreactor scale an immense increase in culturing high cell numbers and elevated quality of cells due to the specific fit of the surface.

## 4. Materials and Methods

### 4.1. Treatment of Polystyrene Materials

Laccase (from Trametes versicolor, Cat. 38429-1G, Lot. BCBR448SV, Merck KGaA, Darmstadt, Germany) was dissolved in PBS (PAA Laboratories, Leonding, Austria) to prepare a working solution of 20 U/mL. Laccase substrates including CA (Cat. NC08.1, Lot. 156243177 CARL ROTH GmbH, Karlsruhe, Germany), L-DOPA (Cat. D9628-5G, Lot. SLBB439V, Merck KGaA, Darmstadt, Germany), and dopamine (Cat. H8502-5G, Lot. BCBS3110, Merck KGaA, Darmstadt, Germany) were prepared at 0.15 μM concentration in PBS. Citrate buffer 2x (SSC) was used to maintain pH 7 (0.3 M sodium chloride, 0.003 M sodium citrate). Non-treated 6-well plates (ThermoFisher Scientific, San Francisco, CA, USA) were processed with laccase and substrate solutions as follows: 80 μL substrate solutions or controls (PBS) were applied to the surface to form a round 11.8 mm diameter treated area, and then 3 μL of laccase working solution was added in droplet form. After 1 h at room temperature, the drops were removed. Treated sites were washed twice with sterile water. Then, 96-well plates were treated accordingly with 100 μL of substrate solution and 3.75 μL of laccase working solution to cover the whole area of the well bottom. Plasma-treated 6-well plates were used as a commercially available control PS surface (ThermoFisher Scientific, USA).

### 4.2. Scanning Electron Microscopy (SEM)

The normal plasma-treated cell culture flasks, untreated flasks, and laccase- and laccase–substrate-treated flasks were rinsed with distilled water, fully dried, and subsequently cut into 8 × 8 mm sections. The plastic squares were mounted onto SEM sample pin mount and sputtered for 30 s at approximately 70 mTorr of pressure to obtain a gold layer with approximately 50 Å thickness. The gold-coated surface of the laccase–substrate treatments was visualized using the scanning electron microscope system (Zeiss SIGMA SEM, Zeiss, Oberkochen, Germany).

### 4.3. Three-Dimensional Surface Scanning

For six selected substrate samples, we measured the surface morphology (texture) using a confocal laser scanning microscope (VK-X1000, Keyence, Osaka, Japan). Surface texture measurements were acquired with a 661 nm laser and a 16-bit CMOS digital camera chip (2048 × 1536 pixel), set to a 50x lens (Nikon OFN25, numerical aperture = 0.8, resolution x,y = 135.032 nm/pixel, step size z = 0.13 µm). The measurements were processed with the software MountainsMap Premium, version 7.48676 (Digital Surf, Besancon, France). We followed the pre-processing filtering procedures for biological surfaces as suggested in [44,45]. The pre-processing filtering procedure consists of the operator’s layer extraction (topography only), outlier removal (excluded: isolated, round edges; normal strength; noise), and filtering (F-filter: least-square leveling; S-filter: Robust Gaussian, order 2, 0.8 µm; L-filter: Double Gaussian, 0.08 mm) and was conducted to achieve the 3D roughness (S-L surface), allowing for the calculation of 11 surface texture parameters. These parameters were selected to quantify roughness as well as possible surface morphology features that could influence cell adhesion (i.e., dales, valleys, hills, summits, pits).

Since the 3D surface texture measurements were not the main focus of this study, we performed three measurements per specimen for illustrative purposes only. Exemplarily, we calculated mean and standard deviation (SD) for one roughness (*Sk*, core roughness depth) and six feature parameters describing the peaks/hills/summits (*Spd*, the density of peaks; *Spc*, arithmetic peak curvature; *Sha*, mean hill area; *Shv*, mean hill volume; *Spk*, reduced summit height), as well as the dales/valleys (*Svd*, the density of pits; *Svc*, arithmetic mean pit curvature; *Sda*, mean dale area; *Sdv*, mean dale volume; *Svk*, reduced valley depth) according to ISO 25178 [41] to illustrate 3D surface roughness. Being aware of the preliminary aspect of the SEM-investigated features, we refrained from further than exemplary illustrative measurements. This viewpoint should be attended to in future studies, since a larger controlled experimental setup would be needed to accurately understand the variation in the dependent variables included. Quantification of surface modifications by surface texture measurements and the statistical modeling using a multilevel multivariate Bayesian model have been previously described in [57].

### 4.4. Cell Culture and Count

For standard cell culture, teratocarcinoma NTERA-2 cells (ACC 527, Leibniz-Institute DSMZ—German Collection of Microorganisms and Cell Cultures GmbH, Braunschweig, Germany) and normal human epidermal melanocytes NHEM (Cat. No., PromoCell, Heidelberg, Germany) were cultured in polystyrene FORMAT cell culture vessels (Merck Life Science Ltd., Darmstadt, Germany) at 37 °C, 5% CO_2_. NTERA-2 were cultured in DMEM (Lonza, Visp, Switzerland) supplemented with 10% FBS (PAA Laboratories, Leonding, Austria) and 2 mM L-Glutamine (PromoCell, Germany). NHEMs were cultured in PCMM medium with the supplement provided by the manufacturer (PromoCell, Heidelberg, Germany). The medium was changed three times a week. Cell confluence was estimated in percent of the populated surface by at least two team members. Cells were subcultured at 70% confluence, using 0.04% trypsin/0.05% EDTA (PromoCell, Heidelberg, Germany). To evaluate the effect of the laccase-mediated surface modification, cells were seeded at a density of xyz in treated 6-well plates and density abc in 96-well plate. Cell adherence was analyzed by estimation of the cell confluence in percent of the populated surface. This estimation was carried out by at least two team members. To determine proliferation, the cells were counted on d xyz using a hemocytometer (for each treatment n = 2).

### 4.5. Mitochondrial Activity Test (WST-1 Assay)

The chromogenic tetrazolium-based WST-1 assay was employed to quantify the mitochondrial activity of NHEM and NTERA-2. Non-treated and plasma-treated 96-well plates were processed with laccase and substrates in a standardized pattern described above. Plasma-treated 96-well plates were used as control. Then, 1 × 10^4^ cells were re-suspended in 200 μL medium, seeded into each well, allowed to adhere for 24 h, and cultivated for up to four days. After the medium was withdrawn, warm PBS was used for washing the cells and subsequently aspirated. Cells were covered with 100 μL of 10:1 DMEM/WST-1 solution per well and incubated for 2.5 h. The WST-1 supernatant was then pipetted and transferred into a flat-bottom 96-well plate. Sample extinction was measured at 450 nm with a 620 nm reference wavelength. The background 620 nm readout was subtracted from that of 450 nm.

We have previously determined a positive association between cell count and the WST-1 chromogenic signal, demonstrating that the alteration in the WST-1 color, which generally presents a display of mitochondrial activity, is directly correlated to the number of melanocytes in the culture. Thereby, WST-1 readout presented a valid criterion for cell proliferation [33,34,35]. This correlation has been confirmed in the methodological setup for this study with both NHEM and NTERA cells (Figure 5). Both total cell count determined by a hemocytometer and mitochondrial activity were used as the measure of the seeded cell adherence on d1 and proliferation measure on d4 of culture.

### 4.6. Melanin Content

Melanin production per NHEM cell was measured as previously reported [33,34,35]. In short, 100,000 NHEMs were seeded into laccase-treated, laccase–substrate-treated, and 6-well plasma-treated PS plates and cultivated for 10 days. NHEMs were harvested, counted, and lysed using 1 N NaOH. The lysate was centrifuged, and the pellet was dissolved in 1 N NaOH and heated for 5 h. The absorbance at OD_475_ was measured and compared against a synthetic melanin curve (Sigma-Aldrich Chemie GmbH, Taufkirchen, Germany) employing linear regression. Thereby, the melanin concentration was retrieved from the sample, with a final readout as the amount of melanin/cell.

### 4.7. Statistical Analyses

Statistical evaluation was performed by means of the unpaired *t*-test performed by Microsoft Office Excel 2016 software. Normal distribution and homogeneity of variance of data sets were checked by the Shapiro–Wilk normality test and F-test. Results with a 95% level of confidence (alpha = 0.05) were referred to as statistically significant.

## 5. Conclusions

The laccase-based surface treatment technology utilized here presents a non-toxic method for increasing adherence properties of the surface that is adjustable in design, easy to use, and inexpensive. The laccase–substrate-mediated PS modification provides a set of surfaces with better adherence properties for the cells. Easier adherence saves resources for the NHEM and NTERA-2 cells and enables reaching the point of sufficient protein and DNA synthesis sooner, which are necessary for the proliferation of cells. The herein reported biofunctionalization of the cell culture surface is highly promising in terms of production optimization.

Moreover, dopamine- and L-DOPA-treated PS surfaces were particularly altered in their geometry and were favorable for adhesion, proliferation, and melanogenesis in melanocytes. Thereby, the potential for cell-type-specific surface modifications by the means of laccase and its substrates has been demonstrated.

This provides an applicative base for custom profiling in biofunctionalization of cell culture surface according to specific cell types and experimental demands. Those remain subject to future work, along with clarification of the cellular processes that mediate surface modifications with enhanced adherence, proliferation, and inherent cell functions upon surface treatment.

## Figures and Tables

**Table 1:** Three-dimensional texture measurements of surface morphology using 3D surface scanning.

Feature	Parameter	Description	Unit	Laccase–Caffeic Acid	Laccase–Dopamine	Laccase–L-Dopamine	Laccase Alone	Plasma	Untreated
Roughness	*Sk*	Core roughness depth	nm	15.086 ± 1.145	16.365 ± 0.257	17.261 ± 0.99	12.831 ± 0.987	11.322 ± 0.212	16.745 ± 0.219
Peaks, hills, summits	*Spd*	Density of peaks	1/µm^2^	0.013 ± 0.002	0.014 ± 0.001	0.009 ± 0.004	0.017 ± 0.003	0.028 ± 0.005	0.018 ± 0.002
	*Spc*	Arithmetic mean peak curvature	1/µm	0.015 ± 0.001	0.017 ± 0.002	0.017 ± 0.001	0.013 ± 0	0.016 ± 0	0.016 ± 0
	*Sha*	Mean hill area	µm^2^	80.854 ± 11.7	73.706 ± 5.519	129.507 ± 67.75	63.294 ± 13.105	37.5 ± 7.531	55.757 ± 5.059
	*Shv*	Mean hill volume	µm^3^	0.038 ± 0.01	0.037 ± 0.002	0.102 ± 0.078	0.02 ± 0.006	0.015 ± 0.004	0.031 ± 0.004
	*Spk*	Reduced summit height	nm	4.687 ± 0.146	6.183 ± 0.69	8.319 ± 3.949	4.665 ± 0.353	3.906 ± 0.235	5.34 ± 0.425
Dales, valleys	*Svd*	Density of pits	1/µm^2^	0.012 ± 0.002	0.014 ± 0.001	0.01 ± 0.004	0.016 ± 0.003	0.029 ± 0.005	0.018 ± 0.002
	*Svc*	Arithmetic mean pit curvature	1/µm	−0.015 ± 0.001	−0.017 ± 0.002	−0.018 ± 0.001	−0.013 ± 0	−0.019 ± 0	−0.018 ± 0.001
	*Sda*	Mean dale area	µm^2^	89.574 ± 14.549	75.911 ± 7.25	123.757 ± 60.314	64.955 ± 11.914	35.708 ± 5.851	56.563 ± 5.336
	*Sdv*	Mean dale volume	µm^3^	0.052 ± 0.007	0.042 ± 0.003	0.099 ± 0.065	0.023 ± 0.006	0.014 ± 0.004	0.039 ± 0.006
	*Svk*	Reduced valley depth	nm	7.283 ± 0.431	7.711 ± 0.65	8.134 ± 1.058	5.278 ± 0.058	5.186 ± 0.36	8.174 ± 0.527

## Data Availability

The data presented in this study are available upon request to the corresponding author.
